# Identifying potential functional impact of mutations and polymorphisms: linking heart failure, increased risk of arrhythmias and sudden cardiac death

**DOI:** 10.3389/fphys.2013.00254

**Published:** 2013-09-20

**Authors:** Benoît Jagu, Flavien Charpentier, Gilles Toumaniantz

**Affiliations:** ^1^INSERM, UMR1087, l'institut du thorax, IRS-UNNantes, France; ^2^CNRS, UMR6291Nantes, France; ^3^Faculté de Médecine, Université de NantesNantes, France

**Keywords:** arrhythmia, sudden cardiac death, cardiac ionic channel, regulation, biogenic properties

## Abstract

Researchers and clinicians have discovered several important concepts regarding the mechanisms responsible for increased risk of arrhythmias, heart failure, and sudden cardiac death. One major step in defining the molecular basis of normal and abnormal cardiac electrical behavior has been the identification of single mutations that greatly increase the risk for arrhythmias and sudden cardiac death by changing channel-gating characteristics. Indeed, mutations in several genes encoding ion channels, such as SCN5A, which encodes the major cardiac Na^+^ channel, have emerged as the basis for a variety of inherited cardiac arrhythmias such as long QT syndrome, Brugada syndrome, progressive cardiac conduction disorder, sinus node dysfunction, or sudden infant death syndrome. In addition, genes encoding ion channel accessory proteins, like anchoring or chaperone proteins, which modify the expression, the regulation of endocytosis, and the degradation of ion channel a-subunits have also been reported as susceptibility genes for arrhythmic syndromes. The regulation of ion channel protein expression also depends on a fine-tuned balance among different other mechanisms, such as gene transcription, RNA processing, post-transcriptional control of gene expression by miRNA, protein synthesis, assembly and post-translational modification and trafficking. The aim of this review is to inventory, through the description of few representative examples, the role of these different biogenic mechanisms in arrhythmogenesis, HF and SCD in order to help the researcher to identify all the processes that could lead to arrhythmias. Identification of novel targets for drug intervention should result from further understanding of these fundamental mechanisms.

## Introduction

Annually, more than 300,000 cases of sudden cardiac death (SCD) occur in the United States, representing a major public health concern (George, 2013). SCD mainly results from severe ventricular arrhythmias, ventricular fibrillation being the most common underlying arrhythmia. These arrhythmias can be the result of a variety of structural changes of the heart or ion channel dysfunctions sometimes through an altered expression. During the last decade, researchers and clinicians have discovered important concepts by elucidating the mechanisms responsible for rare monogenic arrhythmic disorders, so called channelopathies (Martin et al., 2012; George, 2013). Indeed, one major step in defining the molecular basis of normal and abnormal cardiac electrical behavior has been the identification of single mutations that greatly increase the risk for arrhythmias, cardiomyopathies, and SCD (for review see Basso et al., 2011; McNally et al., 2013). Significant technological advances in the study of the genetics of sudden cardiac death have taken place in the last decade (Brion et al., 2010). The vast majority of the mutations identified so far are located in the coding regions of gene encoding ion channel subunits or regulatory proteins and significantly affect the biophysical properties (gating, ion permeation) or the membrane expression of key ion channels. Mouse models of related cardiac arrhythmias, which recapitulate for most of them the clinical phenotypes of the patients, have helped us to elucidate the pathophysiological relevance of these mechanisms (Derangeon et al., 2013; Remme, 2013a). However, patients carrying these mutations remain rare. Heart failure (HF), a syndrome caused by significant impairments in cardiac function, is the leading cause of hospitalization in people older than 65 years in developed countries. HF is a classical situation where the risk of arrhythmias is increased, contributes substantially to morbidity and is responsible for SCD, which represents a large proportion of all deaths in these patients (Janse, 2004; Martin et al., 2011). In HF, and in other cardiac diseases, arrhythmias have been shown to be secondary to electrical remodeling i.e., altered expression of ion channels (Nattel et al., 2010), studied mainly at the mRNA level, although post-transcriptional processes could also be involved (for review see, Zorio et al., 2009; Kim, 2013).

Because of the magnitude of the SCD issue, the opportunities for a real public health impact are significant. SCD is a major health problem and constitutes one of the most important unsolved challenges. Recent medical progresses have had a remarkable impact on SCD in patients at high risk because of advanced heart disease or because they are affected by monogenic arrhythmias (for review, see Martin et al., 2011). However, the majority of victims do not fall into these high-risk groups. Particularly, a significant number of previously healthy young people have died suddenly and unexpectedly, due to genetic heart disorders, either structural cardiomyopathies or arrhythmogenic abnormalities (Brion et al., 2010; Cross et al., 2011). The risk of fatal arrhythmias may be modulated by genetically determined variants in key pathways and may become manifest in the face of environmental triggers such as myocardial ischemia, drugs, or HF (Anvari et al., 2001; Hu et al., 2007a,b; Mittleman and Mostofsky, 2011; Amin et al., 2013). Abnormalities in plasma membrane ion channel function are central to arrhythmogenesis. Recent studies have provided valuable information on how the ion channel expression level, localization, and biophysical properties are regulated, but also revealed that our understanding of the underlying mechanisms is still limited (Harkcom and Abbott, 2010; Ravens and Wettwer, 2011; Rook et al., 2012). Currently, the research should no longer be restricted to study mRNA expression (in acquired cardiac diseases) or biophysical processes (in channelopathies). Although many studies have focused on cell surface expression of mutant channels, it becomes essential to expand our fields of investigation to all the molecular processes responsible for the biogenic properties such as RNA processing, translation and trafficking to understand these fundamental mechanisms. Indeed, incorrect processing causes many diseases. This review focuses on examples chosen to illustrate processes that may account for the malfunction of mutant cardiac channel, allowing to identify novel targets for drug intervention.

## Gene transcription and RNA processing

### Gene transcription and promoter or open-reading-frame polymorphisms

In the last decade, researchers would elucidate inherited arrhythmic disorders by a monogenic approach. But this concept was enable to explain the incomplete penetrance of arrhythmic disorders and the overlap syndromes. These limitations are typically observed in SCN5A-related channelopathies (Remme and Wilde, 2008). The SCN5A gene encodes a voltage-sensitive sodium channel mainly expressed in the cardiac muscle: Nav1.5. Numerous mutations in SCN5A gene have been associated with different rare arrhythmic syndromes, such as type 3 long QT syndrome (LQT3), Brugada syndrome (BrS), cardiac conduction disorders, sick sinus syndrome, atrial standstill and overlap syndromes (Rook et al., 2012). A good example of overlap syndrome is the disease associated with the SCN5A 1795insD mutation. Patients carrying this mutation exhibit bradycardia, conduction disease, LQT3, and Brugada syndromes (Bezzina et al., 1999). Remme and co-workers generated a knock-in mouse carrying the mouse equivalent (1798insD) of the human SCN5A-1795insD mutation and confirmed that a single SCN5A mutation may indeed be sufficient to cause the overlap syndrome (Remme et al., 2006). However, other factors may modify and modulate this clinical phenotype (Remme et al., 2009; Scicluna et al., 2011). These factors, such as the age, gender, drug therapy, associated disease, oligogenic factors, or the genetic background of the patient may explain the diversity of phenotypes observed for the same mutation. The predictive power of autonomic dysregulation and markers such as lipid levels, hypertension, diabetes, and smoking is quite low in subclinical heart disease, the population in which the majority of SCD occur. Thus, it should be considered that a genetic element such as a single nucleotide polymorphism (SNP) could minimize or exacerbate the effect of mutations and must be considered as a functional genetic element. Although the identification of genetic modifiers of disease severity in genetically inherited arrhythmias is rare (Scicluna et al., 2008), the understanding of these diseases will be improved by bioinformatics approaches to identify previously unknown functional genetic elements and to examine their contributions to arrhythmia susceptibility (Arking et al., 2004; Brion et al., 2010; Arking and Sotoodehnia, 2012).

The first level of regulation to study in the “gene to function” relationship remains the level of gene expression that is directly associated with the activity of its promoter region, which plays a central role in the regulation of transcription. In 2006, Bezzina and collaborators identified a set of 6 polymorphisms in near-complete linkage disequilibrium in SCN5A gene promoter region. This variant haplotype, found in about 25% of Asian subjects and absent in whites and blacks, induces a marked reduction of reporter activity in cardiomyocytes (Bezzina et al., 2006). The relationship between SCN5A promoter haplotype and conduction velocity, was analyzed in a cohort of Japanese patients with Brugada syndrome without SCN5A mutations and of Japanese control subjects. This study showed that the variant haplotype was associated with slowed conduction in normal subjects and exacerbated conduction slowing in those with Brugada syndrome. This study provides evidence that genetically determined variable Nav1.5 transcription occurs in the human heart and is associated with variable conduction velocity, an important contributor to arrhythmia susceptibility.

In the same way, Park and collaborators have studied the role of SCN5A promoter variants and DNA methylation by using a family-based approach in predicting phenotype severity in a large kindred with a heterozygous loss-of-function SCN5A mutation (Park et al., 2012). Affected patients exhibited a mixed phenotype of Brugada Syndrome and atrioventricular conduction disease and a marked variation in phenotype severity. During systematic survey of the SCN5A promoter region, they have identified 2 SNP in complete linkage disequilibrium. These promoter variants were significantly associated with disease severity (mild vs. severe phenotype). On the contrary, the analysis of genome-wide DNA methylation profiles did not support a role for the methylation of SCN5A-related genes. This study suggests that the presence of specific promoter variants increase the risk of a severe phenotype in heterozygous carriers of an SCN5A loss-of-function mutation.

These promoter implications on arrhythmogenesis were also confirmed by *in vitro* studies. For instance, Yang and collaborators have identified DNA variants in the proximal promoter region of SCN5A and determined their frequency in 1121 subjects. Interestingly, this population consisted of 88 Brugada syndrome patients with no mutation in SCN5A coding region, and 1033 anonymized subjects from various ethnicities (Yang et al., 2007). Variant promoter activity was assayed in CHO cells and neonatal cardiomyocytes by transient transfection of promoter–reporter constructs. *In vitro* functional analysis identified four variants with significantly reduced reporter activity, up to 62.8% in CHO cells and 55% in cardiomyocytes. The authors concluded that the SCN5A core promoter includes multiple DNA polymorphisms with altered *in vitro* activity, further supporting the concept of interindividual variability in transcription of this gene.

An association between promoter variants and increased arrhythmic risk has also been found for other genes, such as GJA5, which encodes connexin 40. Two closely linked polymorphisms in the promoter of Cx40 gene (−44G→A, rs35594137, and +71A→G, rs11552588) were suggested to decrease Cx40 promoter activity and to be linked to atrial standstill, when expressed homozygously and cosegregating with an SCN5A loss-of-function mutation (Groenewegen et al., 2003), or increased risk of atrial fibrillation (Firouzi et al., 2004). However, Wirka and collaborators have shown more recently that the Cx40 promoter rs35594137 SNP was not associated with altered Cx40 mRNA levels in atria (Wirka et al., 2011). This observation underscores the difficulty of such studies in terms of understanding the molecular basis of cardiac arrhythmias: identifying a SNP in a promoter region is not sufficient, *in vivo* functional validation is necessary. In addition, the authors have identified another common SNP (rs10465885), which alters the configuration of one TATA box of an alternative Cx40 promoter. A promoter-luciferase assay in cultured murine cardiomyocytes demonstrated reduced activity of the promoter containing the minor allele of this SNP. It was strongly associated with Cx40 mRNA expression and displayed strong and consistent allelic expression imbalance in human atrial tissue. It was also associated with early-onset atrial fibrillation.

Several studies have shown an association between angiotensinogen (AGT) promoter polymorphisms and hypertension. For instance, the A-6G and A-20C polymorphisms in the promoter region of AGT gene are associated, respectively, to decreased and increased risks of hypertension (Watkins et al., 2010; Gu et al., 2011). Hypertension is a risk factor for left ventricular hypertrophy, which is a powerful predictor of morbidity and mortality from myocardial infarction, stroke, and congestive heart failure (Rasmussen-Torvik et al., 2005). In the same way, Chen and collaborators have described that the promoter polymorphism G-6A is also associated with non-familial sick sinus syndrome (Chen et al., 2012). This syndrome, including profound sinus bradycardia, sinus arrest, sino-atrial exit block, and tachy-bradycardia, is a group of abnormal heart rhythms presumably caused by a malfunction of the sinus node (Dobrzynski et al., 2007). By *in vitro* approaches, Chen and co-workers have confirmed that nucleotide G at position −6 modulates the binding affinity with nuclear factors and yields a lower transcriptional activity than nucleotide A. The authors concluded that this promoter polymorphism might contribute to non-familial sick sinus syndrome susceptibility.

It should be noted that the vast majority of the mutations identified so far in the context of arrhythmic diseases are located in the coding regions of genes encoding cardiac ion channels or accessory ion channel subunits. These genetic elements can cause life-threatening arrhythmias and sudden death in heterozygous mutation carriers as it has been extensively described for the congenital long QT or Brugada syndromes (for recent review Amin et al., 2013; Remme, 2013b). The application of the same kind of genotype-phenotype relationship between promoter variants and cardiac arrhythmias is a major and critical challenge. Indeed, potential demographic, environmental, and genetic factors in conjunction with a mutation, may modify the phenotype for pathology, and thereby determine, at least partially, the large variability in disease severity.

In this context, some authors have examined the input of promoter mutations under pathological conditions. A deletion/insertion polymorphism (4G/5G) within the gene encoding the plasminogen activator inhibitor 1(PAI-1), has been proposed as a coronary risk factor (Iwai et al., 1998; Margaglione et al., 1998). Indeed, a study performed in healthy people showed that the group with a first-degree relative who had suffered from a coronary ischemic episode had a higher number of homozygotes for the deleted allele (4G/4G) of the PAI-1 gene (Margaglione et al., 1998). Moreover, the 4G/4G and 4G/5G haplotypes have been associated with a faster onset of acute coronary syndromes after the first angina pain (Iwai et al., 1998). Variability of the PAI-1 4G/5G genotype contributes to the variability in circulating PAI-1 levels, with the 4G/4G genotype being associated with higher PAI-1 plasma levels. In this context, Anvari and collaborators have tested the hypothesis that the 4G/4G genotype could promote ischemia-associated malignant ventricular arrhythmias based on the onset of transient coronary ischemic events. They have determined the PAI-1 4G/5G genotypes, as well as PAI-1 antigen levels in 2 groups of patients with coronary artery disease (CAD): one without malignant arrhythmias and one with a history of SCD (Anvari et al., 2001). They revealed a significant association between the 4G allele and the risk for malignant arrhythmias, with greatest risk in subjects possessing the 4G/4G genotype. They also demonstrated that a genetically determined prothrombotic/antifibrinolytic state in patients with CAD may serve as a marker of the severity of the disease, as observed by higher PAI-1 levels in the group of SCD survivors.

In Brugada syndrome, loss-of-function SCN5A mutations have been identified as causative in 20% of cases (Calloe et al., 2013; Remme, 2013b). However, some authors have also examined the input of SCN5A mutations under ischemic conditions. In this context, Antzelevitch and collaborators have enrolled 19 patients developing VF during acute myocardial infarction (AMI) in order to search for possible complications due to SCN5A mutations under ischemic conditions (Hu et al., 2007a,b; Oliva et al., 2009). Among the cohort of 19 patients, one missense mutation (G400A) in SCN5A was detected in a conserved region. An H558R polymorphism was detected on the same allele. Unlike the other 18 patients, who each developed 1–2 VF episodes during AMI, the G400A mutation carrier developed 6 episodes of VT/VF within the first 12 h. This mutation induced a marked decrease in sodium peak current. So they have described the first sodium channel mutation to be associated with the development of an arrhythmic storm during acute ischemia. These findings also suggest that a loss-of-function mutation in SCN5A may predispose to ischemia-induced arrhythmic storm.

These two examples suggest that a genetic variant could serve as a marker of arrhythmic risk in the context of common cardiac diseases.

### Gene transcription and mRNA stability

During transcription, the RNA-polymerase generates long strands of RNA that contain untranslated 5′ and 3′ regions, multiple exons (amino acid encoding RNA sequences) and introns. It should be noted that the functions of non-coding RNA sequences are presently incompletely understood. Regulating the expression of ion channels at the cell surface begins at the level of gene transcription and **mRNA stability**. Thus, pathogenic nucleotide substitutions, deletions, and insertions can affect mRNA synthesis and stability, thereby altering the amount of mRNA available for subsequent protein generation. The 3′- and 5′-UTRs are important in controlling mRNA stability, cellular and subcellular localization, and translation activation or repression (Matoulkova et al., 2012).

Mutations in the human ether-a-go-go-related gene (hERG) result in type 2 long QT syndrome (LQT2). More than 30% of the LQT2 mutations result in premature termination codons. The hERG gene encodes a K^+^ channel that contributes to the repolarization of the cardiac action potential. Gong and collaborators have described that hERG mRNA transcripts that contain premature termination codon mutations are rapidly degraded by nonsense-mediated mRNA decay (NMD) (Gong et al., 2007). The NMD is an evolutionarily conserved RNA surveillance mechanism that recognizes and eliminates transcripts containing Premature Termination Codons (PTC). This process is increasingly recognized as a mechanism for reducing mRNA levels in a variety of human diseases (Nagy and Maquat, 1998; Maquat, 2004; Chang et al., 2007). Gong and collaborators investigated 2 nonsense mutations, W1001X and R1014X, in the C-terminal region of the hERG channel. The primary consequence of the W1001X and R1014X mutations was the degradation of mutant mRNA by NMD. In parallel, the two mutations produced truncated hERG channel proteins and reduced hERG current amplitude. More interestingly, the R1014X mutation also caused a dominant-negative effect on the wild-type hERG channel, which is expected to result in a severe clinical phenotype. Thus, these LQT2 nonsense mutations cause a decrease in mutant mRNA levels by NMD rather than a production of truncated proteins suggesting that the degradation of hERG mutant mRNA by nonsense-mediated mRNA decay is also a significant mechanism in LQT2 patients (Gong et al., 2007). The same mechanism was also described in the pathogenesis of the hERG P926AfsX14 frameshift mutation, which is associated with a severe phenotype (Zarraga et al., 2011).

More recently, Stump and collaborators described another mechanism for the origin of long QT syndrome in which hERG transcripts containing the Q81X nonsense mutation escaped NMD by the reinitiation of translation, resulting in the generation of N-terminally truncated channels. Because the N-terminus of hERG contains several essential regions that contribute to the maintenance of slow channel deactivation, these isoforms exhibited decreased tail current density, accelerated deactivation kinetics, reduced resurgent outward current and co-assembled with wild-type hERG to form heteromeric channels with altered gating properties. The authors present this reinitiation of translation as a new mechanism of hERG channel dysfunction in LQT2 (Stump et al., 2012), in which mRNA stability modulation may induce biophysical changes that contribute to the development of the pathology.

### Alternative splicing

In the post genomic era, it became clear that the number of genes in eukaryotic genomes does not reflect the biological complexity of corresponding organisms. The number of functionally distinct protein isoforms encoded by eukaryotic genes may at least partially explain this lack of correlation. To explain this diversity, it is essential to consider *alternative splicing* as a common eukaryotic process. Normal mRNA splicing can vary for a single gene product, thereby generating multiple transcripts with different coding regions and/or differing translation efficiencies (Kornblihtt et al., 2013). Many splicing variants associated with Nav1.5 and hERG channels have been reported (respectively Shang et al., 2007 and Farrelly et al., 2003). However, the Nav1.5 channel was chosen to illustrate this topic in view of its multiplicity of splicing processes.

**Figure 1:**
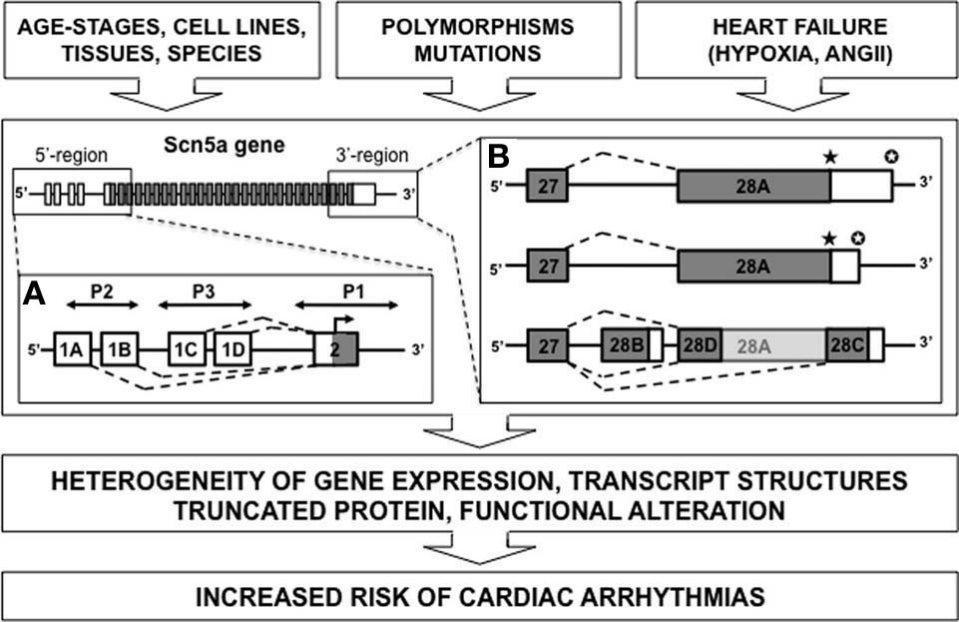
**Diagram depicting alternative splicing for SCN5A gene**. Multiples mRNA variants were described for the 5′ or 3′-untranslated regions (respectively **A** and **B**) (white rectangle, untranslated region; gray rectangle, translated region; *P*, promoter element; ★, stop codon; ✪, polyadenylation site).

In general, SCN5A mRNA is derived from 28 different exons (Figure 1). Exons 2–28 contain the protein-coding sequence, exon 1, and part of exon 2 further encode the 5′-untranslated region (5′-UTR), whereas exon 28 also contains the 3′-UTR. Multiple SCN5A mRNA variants have been detected in the mammalian heart, most of which are generated by alternative splicing and apparently evolutionary conserved mechanisms (Schroeter et al., 2010). Many studies correlated altered action potential morphology and increased arrhythmia vulnerability with changes in the Nav1.5 expression level and/or sodium current (I_Na_) density. Such changes have been frequently reported in common cardiac diseases such as HF (Borlak and Thum, 2003; Valdivia et al., 2005; Shang et al., 2007).

Polymorphisms in the 5′-sequence adjacent to the SCN5A gene have been linked to cardiac arrhythmias. Shang and collaborators have identified three alternative 5′-splice variants (1A, 1B, and 1C) of the untranslated exon 1 in the murine SCN5A mRNA (Shang and Dudley, 2005). Consistent with playing a regulatory role in Na^+^ current, the splice variants differ in relative abundance during development, with a prominent up-regulation of exon 1C in an adult compared with a fetal heart. These 5′-UTRs for SCN5A gene show multiple “Transcription Start Sites” (TSSs). Previously, nine potential TSSs have been identified in rat muscle (Sheng et al., 1994), then three other TSSs have been noted in the human SCN5A gene in sequences preceding the exon 1A (Yang et al., 2004) and finally Shang and collaborators have positioned two other TSSs associated with exon 1C (Shang and Dudley, 2005). Adding the possibility of more complex regulation, the total identified promoter region contained consensus-binding sites for several transcription factors that may be functionally significant. These results suggest the possibility of complex transcriptional and translational regulation of the cardiac sodium channel. Moreover, the SCN5A promoter region is large and complex including repressor elements, tissue-specific promoter elements, and three untranslated exon 1 variants. For instance, the presence of an upstream open reading frame in the human SCN5A transcript containing exon 1D-derived sequences was recently identified. This sequence almost completely abolished translation of Nav1.5 (Van Stuijvenberg et al., 2010). One can easily imagine the pathological consequences of such a complexity.

Many other alternative splicing processes were characterized for this gene (Rook et al., 2012). Among these, there is a splice form in coding region, which is associated with development, knowledge that exceeds the investigation field of arrhythmias.

Nav1.5 possesses a central pore-forming α-subunit, comprising four repeat domains (D1–D4), each composed of six membrane-spanning segments (S1–S6). A major type of alternative splicing of voltage-gated sodium channel genes involves inclusion of two alternative exons 6 (5′ genomic and the 3′ genomic) encoding the D1:S3 segment and the D1:S3/S4 extracellular linker. In developmentally regulated D1:S3 splicing of Nav1.5, there are 31 nucleotide differences between the 5′-exon (“neonatal”) and the 3′-exon (“adult”) forms, resulting in 7 amino acid differences in D1:S3-S3/S4 linker region. Onkal and collaborators (2008) have realized an electrophysiological comparison of “neonatal” and “adult” isoforms. They have observed that the “neonatal” isoform exhibited significant functional differences with the “adult” form. The neonatal isoform presents a shift of steady-state activation toward less negative voltages; it shows a shift of steady-state inactivation toward more negative voltages; has much slower activation and inactivation kinetics; it is associated with significantly greater Na^+^ influx; it shows a stronger voltage dependence of time to peak and recovers from inactivation significantly more slowly (Onkal et al., 2008). In the same time, the authors also tried to determine the critical involvement of a lysine residue in this “neonatal” form. Indeed, they have observed that the K211D mutagenesis in “neonatal” Nav1.5 resulted in a strong shift from the “neonatal” back to the “adult” electrophysiological phenotype. By this approach, they concluded that the negative aspartate (“adult”) to positive lysine (“neonatal”) substitution was primarily responsible for the effects of D1:S3 splicing on Nav1.5 biophysical characteristics (Onkal et al., 2008). Compared to the “adult,” for example, the “neonatal” channel was associated with a larger inward current (Na^+^) during each opening of the channel. In overall conclusion, the authors have stated that electrophysiological characteristics of “neonatal” Nav1.5 are highly likely to have a number of significant pathophysiological as well as physiological consequences. Following this characterization, pathological implications have been attributed to this neonatal form. It was effectively observed that “neonatal” Nav1.5 is a novel marker with significant clinical potential for management of metastatic breast cancer (Onkal and Djamgoz, 2009; Chioni et al., 2010).

Splicing processes within the coding region can also generate truncated forms of Nav1.5. Dudley and collaborators published an interesting example of abnormal Nav1.5 splicing regulation in human HF. This pathological splicing contributes to a reduction in current of a magnitude likely to contribute to the arrhythmic risk in this condition (Shang et al., 2007; Gao et al., 2011). Theses authors have observed that HF results in an increase in two SCN5A mRNA variants, designated Exon 28C (39 bp) and Exon 28D (114 bp). Compared with the full-length Nav1.5 messenger, these variants are shorter and encode prematurely truncated, non-functional Na^+^ channel proteins missing the last part of intramembrane domain IV, from the S3 (for exon 28C) or S4 (exon 28D) segments to the C terminus. The physiological significance of truncations in exon 28 was tested by making a gene-targeted mouse model with a nonsense mutation in this exon between the truncations caused by the E28C and E28D variants. Experiments performed on cardiomyocytes differentiated from embryonic stem cells carrying this mutation at the heterozygous state showed a significant reduction in cardiac Na+ current and conduction velocity (Shang et al., 2007).

But what is the mechanism behind this pathological splicing? During HF, the splicing factors LUC7L3 and RBM25 are up regulated (Choudhary and Dudley, 2002). These proteins are able to bind to the canonical sequence in exon 28 near the splicing sites of SCN5A variants Exon 28C and Exon 28D. This observation has significant pathological implications because the authors also observed that two common features present in HF, Angiotensin II and hypoxia, were able to induce these splicing factors (Gao et al., 2011). This observation was consistent with clinical data suggesting that renin-angiotensin system inhibition and revascularization have antiarrhythmic effects (Moro et al., 2010). This mechanism of splicing does not appear to be tissue restricted but can explain other clinical implications as previously described for cancer (Onkal and Djamgoz, 2009; Chioni et al., 2010).

Are these complex alternative splicing processes characteristic of SCN5A gene or more generally widespread among ion channels? Houtman and collaborators published mapping for two genes encoding the inward rectifier current, KCNJ2 (Kir2.1) and KCNJ12 (Kir2.2) in dog (Houtman et al., 2012). Defective inward rectifier current may lead, amongst other features, to severe cardiac arrhythmias in mouse and man such as ventricular arrhythmias and atrial fibrillation (Anumonwo and Lopatin, 2010). By Race PCR, Houtman and collaborators demonstrated the status of KCNJ2 as a “two exon” gene with a complete Open reading Frame (ORF) in the second exon and only one transcription initiation site was mapped. However, they described four differential transcription termination sites found downstream of two consensus polyadenylation signals. KCNJ12 gene was found to comprise three exons, with its ORF located in the third exon. Only one transcription initiation and one termination site were found for this channel. In addition, the canine KCNJ2 and KCNJ12 gene structures were conserved amongst other vertebrates. Contrary to what has been described for SCN5A, no alternative splicing was observed for KCNJ2 and KCNJ12 genes (Houtman et al., 2012).

Such investigations may be extended to other channels. Thus, mutations in two mutually exclusive exons of the gene encoding the human cardiac L-type calcium channel (Ca_V_1.2) were identified in patients with Timothy syndrome (TS) who exhibit prolonged QT interval and lethal cardiac arrhythmias. Splawski and collaborators have discovered that TS was associated with two Ca_V_1.2 mutations, G406R and G402S. They are located in alternatively spliced exon 8A, encoding transmembrane segment S6 of domain I (Splawski et al., 2005). The spliced form of Ca_V_1.2 containing exon 8 is highly expressed in heart and brain, accounting for approximately 80% of Ca_V_1.2 mRNAs. G406R and G402S cause reduced channel inactivation, resulting in maintained depolarizing L-type calcium currents. These data indicate that gain-of-function mutations of Ca_V_1.2 exons 8 and 8A cause TS. In contrast, the loss-of-function mutations of Ca_V_1.2 channel in patients with Brugada syndrome produce short QT interval that could result in sudden cardiac death (Liao and Soong, 2010). Furthermore, recent reports revealed a linkage of Ca_V_1.2 channel polymorphism with multiple central nervous system disorders including bipolar disorder, depression, and schizophrenia.

Nevertheless, the channels are not the only cases of genetic variability affecting gene splicing described to date. Thus, Refaat and collaborators have worked on the prevalence of mutations in the RNA splicing protein RBM20 in a large cohort of patients with dilated cardiomyopathy (DCM) (Refaat et al., 2011). The coding region and splice junctions of RBM20 were screened in subjects with DCM. Following this research, 2 common polymorphisms in this splice factor, rs942077 and rs35141404, were genotyped in all subjects. Although mutations in RBM20 were observed in approximately 3% of pathological subjects, no differences in survival, transplantation rate, and frequency of ICD therapy in mutation carriers were observed. Despite this apparently negative result, such studies should be generalized and extended to all the proteins involved directly or indirectly in the mechanism of splicing.

## Translational control by miRNA and alternative translation

Improving our knowledge on molecular mechanisms of cardiac arrhythmias will also necessitate gaining knowledge on post-transcriptional mRNA regulation.

### Translational control by miRNA

Noncoding RNA sequences, with incompletely understood function, appear to be involved in the post-transcriptional regulation by microRNAs (miRNA). The complexity of this regulation is still not completely understood and should represent a novel concept about combination of basic research and clinical application.

**Figure 2:**
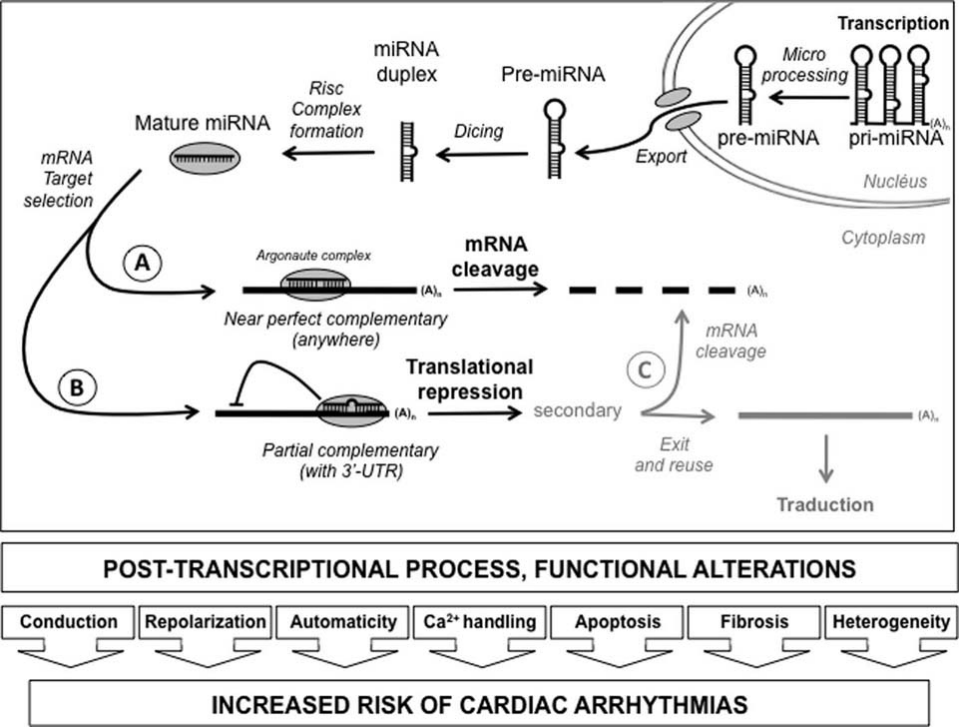
**Diagram depicting post-transcriptional process by miRNA**. After their transcription, the pri-miRNA were matured in pre-miRNA and exported to cytoplasm. After digestion by dicer and Risc complex formation, the mRNA target selection can take place depending on the quality of the match. When the complementary is near perfect, throughout the length of the mRNA, cleavage of the messenger will take place leading to its degradation **(A)**. However, if the setting is only partial, usually in the 3′-untranslated region, a translational repression can take place without any degradation on the messenger **(B)**. Secondarily, it is possible for the cell to remove the inhibition and to reuse either definitively destroy the messenger **(C)** [*3′-UTR*: 3′-untranslated region; (A)_n_: polyadenylation tail].

MiRNAs are small noncoding RNAs that regulate the expression of target 1) by a direct degradation of their target mRNA following a near-perfect hybridization, but this case is rare or 2) by binding to sequence that include the 3′ untranslated region (3′-UTR) of newly synthesized mRNA transcripts by a block of translation (Figure 2). The human genome is estimated to encode more than 1000 miRNAs, which are either transcribed as stand-alone transcripts, often encoding several miRNAs, or generated by the processing of protein coding gene introns. The miRNAs typically exert their inhibitory effects on several mRNAs, which often encode proteins that govern the same biologic process or several components of a molecular pathway.

Although published studies focusing on miRNAs and cardiac excitability are still sparse, recent articles have highlighted the role of miRNAs in cardiac rhythm through regulation of key ion channels, transporters, and cellular proteins in arrhythmogenic conditions (Kim, 2013). The available data from experimental studies demonstrate that miRNAs regulate numerous properties of cardiac excitability including conduction, repolarization, automaticity, Ca^2+^ handling, spatial heterogeneity, and apoptosis and fibrosis. To illustrate this post-transcriptional process, we have chosen to discuss about two suitable examples.

Drawnel and collaborators have described an interesting mutual antagonism between inositol 1,4,5′-triphosphate receptor II (IP_3_RII) calcium channel and miRNA-133a. This antagonism regulates calcium signals and cardiac hypertrophy (Drawnel et al., 2012). Indeed, IP_3_RII expression is increased in hypertrophic failing human myocardium. The ectopic calcium released from these receptors induces pro-hypertrophic gene expression and may promote arrhythmias. Drawnel and collaborators have investigated the mechanisms that produce up regulation of IP3RII during hypertrophic cardiomyocyte remodeling. This study clearly delineates an anti-hypertrophic role for miR133a that is intrinsic to the ventricular myocyte by maintaining low basal IP3RII expression. The authors have observed that decreased expression of miR-133a allows IP3RII expression to increase, thereby promoting hypertrophy. This maintains an appropriate level of myocyte growth until the balance between miR-133a and IP3RII is perturbed by pathological stimuli that elicit calcium signals. The authors have also described a deleterious positive feedback loop. Indeed, an increase in calcium signals sustains repression of miR-133a, providing a powerful driving force for pathological remodeling. In a relevant manner, other pro-hypertrophic positive feedback loops have been identified involving miRNAs and the signaling pathways that regulate their expression. This is the case for miR-1–dependent modulation of insulin-like growth factor-1 signaling (Elia et al., 2009) and miR-199b–mediated regulation of dual-specificity tyrosine phosphorylation-regulated kinase 1a (Dyrk1a), the NFAT negative regulatory kinase (da Costa Martins et al., 2010). When taken together, these studies indicate that several reciprocal regulatory loops may be active within the myocardium under pro-hypertrophic conditions. The potential clinical implications are interesting. Indeed, miR-133a activity *in vivo* is sufficient to mediate pathological cardiac growth. In particular, infusion of miR-133a antagomir (a miRNA blocker) is sufficient to induce hypertrophic remodeling in adult mice (Carè et al., 2007). However, things remain to be clarified as other authors have observed that miR-133a maintains cardiac function via anti-apoptotic and anti-fibrotic effects within the adult myocardium (Matkovich et al., 2010). But this observation does not take away the relevance of this mode of regulation in our arrhythmia issue.

To illustrate the cardiovascular consequences of this post-transcriptional regulation, we will focus on Friedreich ataxia (FRDA), which is an autosomal recessive neurodegenerative disease arising from mutations in both alleles of the frataxin gene (FXN). In approximately 97% of cases the mutant alleles have an expansion of a GAA trinucleotide repeat in intron 1 of FXN that reduces the amount of frataxin available to assist with mitochondrial iron efflux and increases sensitivity to oxidative stress, resulting in cell damage and death due to excess production of free radicals (Cossee et al., 1997; Wong et al., 1999). As well as the neurological features of the disease, a large proportion of FRDA patients develop cardiac abnormalities of cardiac structure or function. Indeed, heart failure and cardiac arrhythmias are thought to be the most important causes of death in FRDA (De Michele et al., 1996).

Kelly and collaborators have used a tag SNP approach to investigate the association of genetic variability in three important genes of the Renin–Angiotensin–Aldosterone system (RAAS) with measures of cardiac phenotype severity in FRDA (Kelly et al., 2011). The genes selected for investigation were AGTR1, which encodes AT1R, the predominant angiotensin-II receptor in the cardiovascular system, and the ACE and ACE2 genes, which encode the converting enzymes directly involved in the production and degradation of angiotensin-II. On the basis of this investigation, the authors have identified rs5186, a SNP in AGTR1, as a potential modifier of the cardiac phenotype in a large population of FRDA patients. The rs5186 SNP is a functional A/C polymorphism that occurs in the target sequence of a regulatory microRNA, miR-155, in the 3′ untranslated region of AGTR1 (Martin et al., 2007). However, the rs5186 C allele interrupts base-pair complementarily between miR-155 and the cis-regulatory target site, decreasing the ability of miR-155 to bind, thereby increasing the expression of AT1R (Kelly et al., 2011). The unexpected and intriguing finding of this study was that the C allele of rs5186 was more common in this cohort of FRDA patients than in a healthy control population. Further investigation of the prevalence and cardiovascular effects of rs5186 in other large FRDA populations is required, but it appears that this polymorphism should present a protective effect, resulting in increased survival. Indeed, the prohypertrophic cardiac effects assigned to this polymorphism, confer a survival advantage for FRDA patients that present this SNP. Future studies have to assess the activity of miR-155 in FRDA patients, as it is the likely mechanism by which rs5186 modifies the cardiac phenotype, but these observations underline the importance of this mode of regulation in cardiovascular remodeling. Despite many recent article withdrawals, the current literature on miRNAs is rich and alterations of this mode of post-transcriptional regulation need to be systematically studied in our investigations of arrhythmia mechanisms.

### Alternative translation

Another molecular mechanism potentially allowing a single mRNA to produce several proteins is alternative translation (for review, see Kochetov, 2008).

It is widely established that an eukaryotic mRNA typically contains one translation start site and encodes a single functional protein product. In mammalian translation systems the consensus sequence is “GCCRCCAUGG” in positions near the start AUG codon (Kozak, 2005). However, it is now well-documented that a single mRNA may code for multiple proteins based on utilization of alternative initiation codons. “Non-AUG codons,” as well as the classical AUG start codon, should be jointly considered in mechanisms for translation of mRNAs. These “non-AUG codons” are represented by the codons CUG, GUG, UUG, AUA, and ACG. These alternative Translation Initiation Sites (aTIS) give new opportunities to the cell for their protein synthesis by increasing the number of functionally novel protein isoforms encoded by genes, which present non-AUG codons. However, open-reading frames started from alternative start codons can commonly be located in different positions. The proteins resulting from these aTIS can be unrelated. Nevertheless, in most cases, the “non-AUG codons” respect the main ORF and lead to the synthesis of either N-end truncated or N-end extended isoforms of the CDS-encoded protein, commonly rather small. At the sight of this new knowledge, polymorphisms or mutations located within or downstream of the canonical ATG codon of candidate sequences should be studied with renewed interest.

It is well-established that the N-end segment of proteins frequently contains secretory signals. Thus, these isoforms can differ in the protein N-end segment and may be delivered to different compartments and present different subcellular localizations. In this context, this process takes all its full meaning for channel trafficking. One should nevertheless be cautious about any rapid conclusion. Indeed, channels have the added complexity of possessing several transmembrane spanning domains with numerous signal sequences, not restricted to the amino terminal, to be targeted to plasma membrane. For example, Lu and collaborators have described that multiple topogenic determinants cooperate during Kv1.3 voltage-gated K^+^ channel translocation. Only the transmembrane segment S2 likely functions as the initial signal sequence to determine Kv1.3 N-terminus topology (Tu et al., 2000). Thus, the changes in the N-terminus portion of a given channel by aTIS cannot always explain alone the channel trafficking defect. So in this context, aTIS identification must be taken into account with caution on an individual basis according to the considered channel.

Numerous mammalian regulatory proteins utilize aTIS as critical regulators that control distinct biological functions like metabolism, intracellular signal transduction, transcription and gene expression, growth mechanisms, and related cellular functions (Kochetov, 2008). And the number of experimentally verified examples of alternative translation is growing rapidly.

Nevertheless, it should be noted that alternative translation initiation is a rare process in mammalian mRNA. In this context, bioinformatics evaluations of 5′-UTR sequences of mammalian mRNAs will represent a critical opportunity to characterize new aTIS in mRNA sequences that have been experimentally involved in pathologies. In the field of ion channels, a computational analysis of aTIS performed by Wegrzyn and collaborators a few years ago suggested that the calcium channel γ8 ancillary subunit (CACNG8) contains at least one alternative initiation site (Wegrzyn et al., 2008). This protein was recently identified as a regulator of the main human heart L-type calcium channel, Ca_V_1.2 (Yang et al., 2011). Other genes involved in cardiovascular functions, such as the Vascular Endothelial Growth Factor (VEGF) have also been shown to contain aTIS (Wegrzyn et al., 2008). The ability to define aTIS through computational analyses can be of high importance for genomic analyses to provide full predictions of translated mammalian and human gene products required for cellular functions in health and disease. Whether aTIS plays a role in cardiac electrical activity and more broadly in cardiovascular physiopathology remains an open question.

## Post-translational modifications and trafficking

A recent bibliography reflects quite well this mode of regulation. Post-translational modification of cardiac ion channels is a cellular mechanism for maintaining the rhythmicity of the heartbeat. Therefore, several studies have made it clear that extensive post-translational modifications may modulate cardiac channel expression levels, localization, and gating.

The glycosylation is one of the most common post-translational modifications. The Nav1.5 protein harbors multiple evolutionary conserved amino acid motifs for N-glycosylation in its extracellular domain. Although the precise molecular composition of the N-glycans in cardiomyocytes and their attachment sites have not been determined yet, their presences in cardiac Nav1.5 and impact on channel gating have been recognized. Thus, Zang and collaborators have described that glycosylation influences voltage-dependent gating of cardiac and skeletal muscle sodium channels (Zhang et al., 1999). The contribution of sugar residues to channel gating was examined in transfected cells pretreated with various glycosidase and enzyme inhibitors to deglycosylate channel proteins. Pretreating transfected cells caused depolarizing shifts of steady-state activation of hH1a (human isoform of Nav1.5). These data clearly suggested that glycosylation differentially regulates sodium channel function in heart and skeletal muscle myocytes. The same observation was done for the adult rat ventricular myocytes (Stocker and Bennett, 2006). In this context, Johnson and collaborators have described interesting observations on the glycosylation of β-1 subunit of voltage-gated sodium channels (Nav) (Johnson et al., 2004). Nav are composed of a pore-forming alpha subunit and often one to several modulating beta subunits. The fully sialylated ß1 subunit induces a uniform hyperpolarizing shift in steady state and kinetic gating of the cardiac and two neuronal alpha subunit isoforms. Moreover, Johnson and collaborators have observed that under conditions of reduced sialylation, the ß1-induced gating effect was eliminated. These observations are consistent with the fact that the mutation of ß1 N-glycosylation sites abolished all effects of ß1 on channel gating. Thus, as it has been shown that glycosylation could differ according to the location within the heart tissue (Montpetit et al., 2009) and as electrical remodeling in cardiac disease is usually ascribed to altered expression and distribution of ion channel proteins such as Nav1.5, the location of any mutation in these consensus sites of modification should be considered as probable causes of arrhythmia occurrence.

This type of argument can obviously be extended to other channels. Chandrasekhar has recently described that an essential regulatory subunit of the cardiac I_Ks_ potassium channel complex, KCNE1, is glycosylated at threonine-7 *in vivo* (Chandrasekhar et al., 2011). Mutations that prevent glycosylation at this amino acid result in I_Ks_ channel complexes that are unable to efficiently traffic to the plasma membrane. Indeed, examination of these mutants revealed that complexes that lack N-terminal glycans adjacent to the N-terminus were functionally similar to wild type (WT), but had significantly reduced cell surface expression. Thus, mutations on threonine-7 directly suppress the O-glycosylation site and have a dramatic effect on biogenesis and anterograde trafficking of the protein complex, yielding unglycosylated and mono-N-glycosylated complexes that are trafficking defective and compromised, respectively. These observations provide a cellular mechanism for a KCNE1 mutation on threonine-7 that may be associated with cardiac arrhythmias.

The glycosylation is a critical regulation, but other types of post-translational modifications are not to be neglected, such as phosphorylation. In this context, Nav1.5 and ß-adrenergic receptors colocalize to caveolin domains that participate in membrane trafficking. Yarbrough and collaborators have shown that under stimulation of beta-adrenergic receptors, a mechanism involving the alpha subunit of the stimulatory heterotrimeric G-protein, Galpha(s), promotes the presentation of cardiac sodium channels associated with caveolar membranes to the sarcolemma, leading to an increase in sodium current amplitude (Yarbrough et al., 2002). In addition, Zhou and collaborators have demonstrated that the activation of cAMP-dependent protein kinase (protein kinase A) secondary to the stimulation of beta-adrenergic receptors can potentiate I_Na_ by two processes: a fast saturable and a slow unsaturable component. The fast component involves direct channel phosphorylation events regulating the kinetics and voltage dependence of channel gating, while the slow component of PKA-dependent I_Na_ potentiation is due to enhanced trafficking and insertion of additional functional channels into the membrane. It should be noted that the I-II cytoplasmic interdomain linker loop is critical for this effect (Zhou et al., 2000). However, other critical sites have been characterized. The I-II interdomain linker of the channel contains 3 sites with the RXR motif known to mediate retention of proteins in the endoplasmic reticulum. The PKA-mediated increase in Na^+^ current was completely abolished when all 3 sites were eliminated (Zhou et al., 2002). These results demonstrate that both phosphorylation and the presence of putative ER retention signals are required for the PKA-mediated increase in cardiac Na^+^ current. These observations may have important physiological consequences for the increase in cardiac conduction velocity observed with sympathetic stimulation and the genesis of re-entrant arrhythmias in ischemic myocardium. Herren and collaborators focussed on the molecular and functional aspects of Na^+^ channel phosphorylation, which is potentiated in heart failure and has been causally linked to cardiac arrhythmias (Herren et al., 2013).

The oxidation/nitrosylation is also one of the critical and ubiquitous post-translational modification systems for the regulation of cardiac ion channels (for reviews Gonzalez et al., 2009; Herren et al., 2013). S-nitrosylation consists of the addition of a nitric oxyde (NO) group to the thiol moiety of a cysteine residue. Several ion channels are reportedly redox responsive. For example, Xu and collaborators have explored the mechanism of NO action on the cardiac calcium release channel, of the sarcoplasmic reticulum, the type 2 ryanodine receptor (Xu et al., 1998). They have described that the S-nitrosylation led to progressive channel activation, which was reversed by denitrosylation. In contrast, its oxidation had no effect. So this channel can differentiate nitrosative from oxidative signals. The authors suggest that NO and related molecules may regulate excitation-contraction coupling through discrete mechanisms. On the one hand, they can inhibit the L-type channel via cGMP; on the other hand, they sensitize the muscle to Ca^2+^-induced Ca^2+^ release. So the cardiac ion channels subserving excitation-contraction coupling are potentially regulated by S- nitrosylation. In the same way, the sodium channel is rich in cysteine and the metabolic state is also intimately coupled to sodium current. However, this process appears to have tissue specificity. Indeed, a full S-nitrosylation motif of the acid base type is found in the Nav of sensory neurons, but only partial in the heart (Li et al., 1998). As for the ryanodine receptor, it is possible that beside S-nitrosylation, Nav channels could be regulated also by cysteine oxidation (Evans and Bielefeldt, 2000). Ueda and collaborators have described an association of this post-translational regulation with inherited long QT syndrome (Ueda et al., 2008). They have identified a missense mutation by direct sequencing of the gene encoding alpha1-syntrophin (SNTA1), a member of the dystrophin-associated proteins normally serving as a scaffold protein for the neuronal nitric oxide synthase (nNOS) and the plasmalemmal calcium pump PMCA, an interaction that results in inhibition of NO production. The SNTA1 is also known to associate with Nav1.5. Syntrophin mutation results in a disruption of the PMCA-NOS1 complex and favors interaction of NOS1 with the Na^+^ channel. Release of PMCA increases NOS1 activity promoting S-nitrosylation of Nav1.5 and thereby increasing late Na^+^ currents at the origin of LQTS-susceptibility.

Another interesting mechanism is the potential dominant-negative effect due to a heterozygous expression of a mutated form. Type 2 long QT syndrome involves mutations in the human ether a-go-go–related gene (hERG or KCNH2) (Keller et al., 2005), which encodes Kv11.1 channel. Kv11.1 channel current, because of its unique slow activation and deactivation gating kinetics relative to its rapid kinetics of inactivation and recovery from inactivation, plays a significant role in late repolarization in the mammalian heart. As T421M, a mutation in the S1 transmembrane spanning domain of Kv11.1, was identified in a resuscitated patient, Balijepalli and co-authors have assessed its biophysical, protein trafficking, and pharmacological consequences in adult rat ventricular myocytes (Balijepalli et al., 2012). The T421M mutation markedly altered the voltage dependence and kinetics of both Kv11.1 activation and deactivation but had minimal effects on the rates of inactivation and recovery from inactivation. Furthermore, interestingly, for coexpressed wild-type and T421M-Kv11.1 channels, different dominant-negative interactions govern protein trafficking and ion channel gating, and these are likely to be reflected in the clinical phenotype.

Malfunction may also result from defects in the multiprotein machinery specialized in channel membrane targeting. In this context, Wu and collaborators have described MOG1, a small protein that is highly conserved from yeast to human, as a critical co-factor for Nav1.5. This protein, expressed in both atrial and ventricular tissues with predominant localization at the intercalated discs, can modulate the function of Nav1.5 (Wu et al., 2008). MOG1, which interacts physically with Nav1.5, increases sodium current density by an increase in the number and/or availability of Nav1.5 on cell surface. In addition to increased trafficking of Nav1.5 to the plasma membrane, MOG1 may reduce the turnover of Nav1.5 localized on the plasma membrane.

Thus, genetic mutations in MOG1 may affect the expression and function of Nav1.5, leading to Brugada syndrome or other types of lethal arrhythmias. Indeed, Kattygnarath and collaborators have reported by genetic screening that the MOG1 missense mutation E83D could affect Nav1.5 activity and provide molecular and clinical evidence that this MOG1 loss-of-function mutation is linked to Brugada syndrome physiopathology (Kattygnarath et al., 2011). As expected, this mutant exerted a dominant-negative effect on wild-type MOG1 and reduced Nav1.5 channel trafficking to the cell surface. Similarly, Olesen and collaborators have screened MOG1 for variants in 197 young patients with lone atrial fibrillation and 23 patients with Brugada syndrome and identified a novel nonsense variant mediating a premature stop codon, p.E61X (Olesen et al., 2011). Their heterologous expression data on p.E61X mutant showed MOG1 loss of function. This mutant completely failed to increase the sodium channel current compared to wild-type MOG1. Nevertheless, this variant seemed to have a higher frequency in patients compared to control subjects but the difference was not statistically significant. Also in this context, Chakrabarti and collaborators have recently hypothesized that MOG1 can serve as a therapeutic target for sodium channelopathies. They have observed that this co-factor can enhance plasma membranes trafficking of mutant sodium channels and rescue the reduced sodium current associated with Nav1.5 mutations leading defects in trafficking (Chakrabarti et al., 2013). These results indicate that MOG1 could also be a potential Brugada syndrome modifier gene and could explain part of the variable penetrance of the pathology. MOG1 is not the only example of modulation of trafficking for Nav1.5 by a chaperone protein. For example, Ishikawa and collaborators have found that two missense mutations in the gene encoding the sarcolemmal membrane-associated protein (SLMAP), Val269Ile and Glu710Ala, affect Nav1.5 membrane surface expression (Ishikawa et al., 2012). These mutations in SLMAP, which ultimately reduce Nav1.5 current, may cause Brugada syndrome via modulating the intracellular trafficking of Nav1.5.

These defaults of trafficking caused by mutations have been described for many other cardiac ion channels, including KCNQ1 potassium channel, Sato and collaborators have investigated the functional alterations caused by 2 KCNQ1 mutations, a deletion (delV595) and a frameshift (P631fs/19), which were identified in patients with autosomal-recessive LQTS not accompanied by hearing loss (Sato et al., 2009). Functional analyses showed that both mutations impaired cell surface expression due to trafficking defects. The mutations severely affected outward potassium current. It was found that delV595 impaired the binding and assembly of KCNQ1 subunits, whereas the P631fs/19 mutant channel was retained in endoplasmic reticulum due to the newly added 19-amino acid sequence containing two retention motifs (R(633)GR and R(646)LR. In the same manner, a channel may present multiple kinds of abnormalities. Limberg and collaborators have identified two novel heterozygous KCNJ2 mutations (p.N318S, p.W322C) located in the C-terminus of the Kir2.1 potassium channel subunit in a large set of patients with congenital long-QT syndrome (Limberg et al., 2013). While the N318S mutants regularly reached the plasma membrane, W322C mutants primarily resided in late endosomes. The co-expression of N318S or W322C with wild-type Kir2.1 reduced current amplitudes by 20–25% due to defective channel trafficking (W322C) or gating (N318S). Thus, understanding the dynamics of cardiac channel surface expression and their potential trafficking abnormality has become even more essential.

## Conclusions

Thus, at the sight of the multiplicity of mechanisms regulating ion channel protein expression, such as gene transcription, RNA processing, post-transcriptional control of gene expression by miRNA, protein synthesis, assembly and post-translational modification and trafficking, polymorphisms possibly affecting these mechanisms should be investigated in our work of understanding new processes at the origin of arrhythmogenesis, not only as phenotype modulators of genetically-inherited arrhythmias, but also as putative a arrhythmic substrates in more common diseases such as cardiac hypertrophy and heart failure.

### Conflict of interest statement

The authors declare that the research was conducted in the absence of any commercial or financial relationships that could be construed as a potential conflict of interest.
